# Multi-omic approach identifies hypoxic tumor-associated myeloid cells that drive immunobiology of high-risk pediatric ependymoma

**DOI:** 10.1016/j.isci.2023.107585

**Published:** 2023-08-09

**Authors:** Andrea M. Griesinger, Kent Riemondy, Nithyashri Eswaran, Andrew M. Donson, Nicholas Willard, Eric W. Prince, Simon M.L. Paine, Georgia Bowes, John Rheaume, Rebecca J. Chapman, Judith Ramage, Andrew Jackson, Richard G. Grundy, Nicholas K. Foreman, Timothy A. Ritzmann

**Affiliations:** 1 Morgan Adams Foundation Pediatric Brain Tumor Research Program, Children’s Hospital Colorado, Aurora, CO 80045, USA; 2 Department of Pediatrics, University of Colorado Denver, Aurora, CO 80045, USA; 3 Colorado Clinical and Translational Sciences Institute, University of Colorado Denver, Aurora, CO 80045, USA; 4 RNA Bioscience Initiative, University of Colorado Denver, Aurora, CO 80045, USA; 5 Department of Pathology, University of Colorado Denver, Aurora, CO 80045, USA; 6 IsoPlexis, Branford, CT 06405, USA; 7 Department of Neurosurgery, University of Colorado Denver, Aurora, CO 80045, USA; 8 Children’s Brain Tumour Research Centre, University of Nottingham Biodiscovery Institute, Nottingham, UK; 9 University of Nottingham Biodiscovery Institute, Nottingham, UK; 10 Nottingham University Hospitals NHS Trust, Queen’s Medical Centre, Derby Road, Nottingham NG7 2UH, UK

**Keywords:** Microenvironment, Biopsy sample, Components of the immune system, Proteomics, Transcriptomics

## Abstract

Ependymoma (EPN) is a devastating childhood brain tumor. Single-cell analyses have illustrated the cellular heterogeneity of EPN tumors, identifying multiple neoplastic cell states including a mesenchymal-differentiated subpopulation which characterizes the PFA1 subtype. Here, we characterize the EPN immune environment, in the context of both tumor subtypes and tumor cell subpopulations using single-cell sequencing (scRNAseq, n = 27), deconvolution of bulk tumor gene expression (n = 299), spatial proteomics (n = 54), and single-cell cytokine release assays (n = 12). We identify eight distinct myeloid-derived subpopulations from which a group of cells, termed hypoxia myeloid cells, demonstrate features of myeloid-derived suppressor cells, including IL6/STAT3 pathway activation and wound healing ontologies. In PFA tumors, hypoxia myeloid cells colocalize with mesenchymal-differentiated cells in necrotic and perivascular niches and secrete IL-8, which we hypothesize amplifies the EPN immunosuppressive microenvironment. This myeloid cell-driven immunosuppression will need to be targeted for immunotherapy to be effective in this difficult-to-cure childhood brain tumor.

## Highlights

• Identification of eight distinct pediatric ependymoma-infiltrating myeloid cells
• Cellular fractions influence subgroup specific-phenotypes in bulk tumor expression
• Infiltrating immune cells border areas of tumor necrosis and perivascular niches
• Myeloid suppressor features of hypoxia myeloid cells drive PFA ependymoma biology

## Graphical abstract

**Figure:**
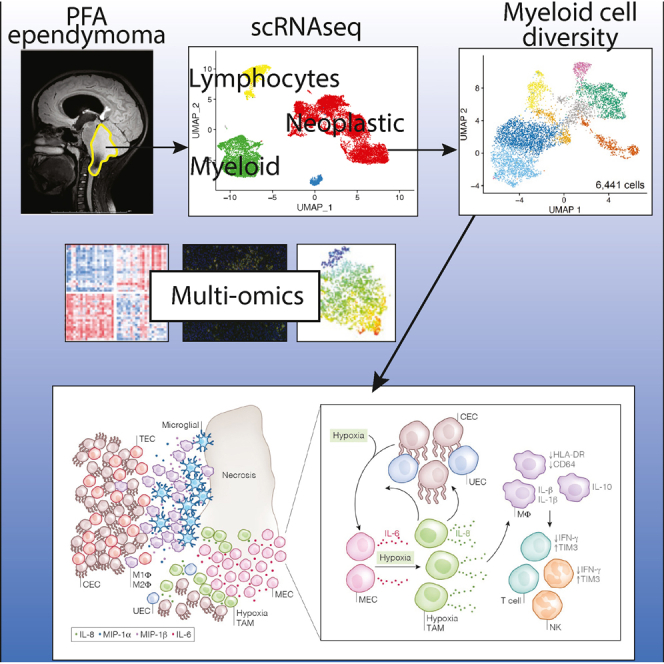

## Introduction

Ependymoma (EPN) is a major cause of cancer-related death in childhood and adolescence. Relapse occurs in 50% of children, of whom only 25% survive beyond five years.^1^^,^^2^ Survival has shown only modest improvements despite modern, standard-of-care therapy.^3^^,^^4^^,^^5^^,^^6^ Ground-breaking advances in molecular classification have identified children with posterior fossa A ependymoma (PFA) as having the worst outcomes.^3^^,^^7^ This lack of progress highlights the need for a paradigm shift away from conventional, non-targeted, therapies toward a deeper understanding of the underlying tumor biology to facilitate a more rational and effective approach to treatment.

Given previously described associations between immune factors and EPN, immunotherapy trials, including those using CAR-T cell technology, have commenced.^8^ While adoptive cell therapies have potential benefits in this disease, pediatric PF EPN harbor a lack of coding mutations alongside evidence of phenotypically and functionally exhausted host lymphocytes, further restricted by an immunosuppressive tumor microenvironment. All these factors may culminate in failure to establish an antitumor T cell cytolytic response.^9^^,^^10^^,^^11^

While knowledge of the underlying EPN immunobiology is evolving, there remains a lack of detailed knowledge of the complexity of its immunosuppressive microenvironment which in turn risks hampering the development of urgently required new therapies.^12^^,^^13^

Prior work has shown an association between antitumor immune gene signatures and myeloid cell function with improved survival^9^^,^^12^^,^^13^^,^^14^^,^^15^^,^^16^ and a strong correlation between immune gene signatures and molecular subgroups of posterior fossa EPN (PFA1 and PFA2).^5^^,^^9^ PFA1, the largest and most aggressive subgroup, has a gene signature associated with inflammation, hypoxia, and angiogenesis, and has a particularly poor clinical outcome. Conversely, PFA2 has a more favorable antitumor immune signature featuring genes associated with an antiviral response.^9^ Functional studies have shown that PFA1 tumor cells reprogram myeloid cells to a myeloid-derived suppressor cell (MDSC)-like phenotype through an NF-κB/IL-6/STAT3 signaling pathway.^9^^,^^12^^,^^13^

Recent EPN single-cell sequencing studies have provided novel insights into intra-tumor heterogeneity.^17^^,^^18^ Gillen and colleagues identified four major PFA neoplastic cell types.^17^ PFA1 EPNs were enriched for mesenchymal EPN cell (MEC) populations found specifically in necrotic and perivascular niches. PFA2 EPNs were enriched for ciliated EPN cells (CEC), consistent with cilia-related ontologies seen in bulk gene expression profiling of PFA2 and posterior fossa B EPN. Tumor-infiltrating immune cells were also described by Gillen et al. but only generically defined as the focus for that study was neoplastic cell heterogeneity. Subsequently, the advent of spatial transcriptomics has facilitated better delineation of the spatial implications of the single-cell sequencing studies, with evidence that multiple neoplastic and immune cells interact in the process of moving cells through spatially restricted hierarchies of epithelial-mesenchymal transition, populated by a pool of undifferentiated EPN cells (UEC), facilitating the process of tumor progression and maintenance.^19^ However, spatial transcriptomics currently lacks the single-cell resolution necessary to fully characterize the immune cells, necessitating other approaches.

Here, we provide an in-depth analysis of the PFA EPN immune environment, focusing directly on the heterogeneity of the myeloid cell subpopulations, using single-cell and bulk tumor transcriptomics, single-cell spatial proteomics, and single-cell cytokine/chemokine secretome assays. We report a hypoxia myeloid subpopulation with features of myeloid-derived suppressor cells and provide data to support the hypothesis that the interaction between the MEC neoplastic subpopulation and the hypoxia myeloid subpopulation is a key driver of the PFA1 immunosuppressive microenvironment.

## Results

### Single-cell RNA sequencing identified 8 subpopulations of EPN tumor-infiltrating myeloid cells

**Figure 1:**
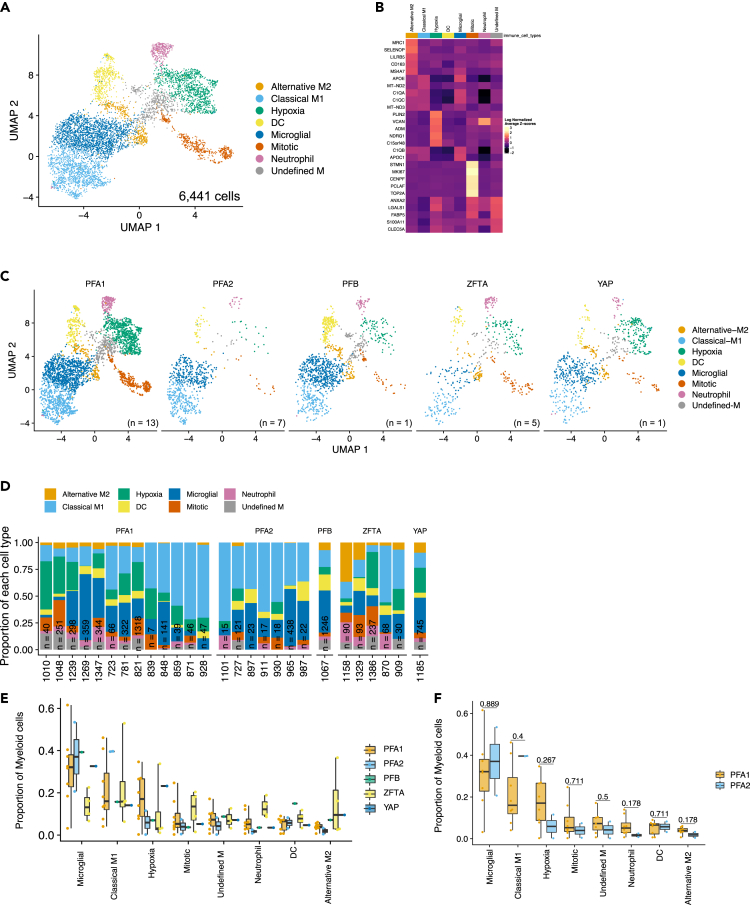
scRNAseq analysis of immune subpopulations in EPN reveals 8 unique subpopulations of myeloid cells We sequenced 26 viably frozen, single-cell suspensions, of pediatric ependymoma with a minimum of 2000 cells per samples. (A) UMAP clustering of myeloid cells identified 8 transcriptionally unique subpopulations of cells. Populations were named based on the gene expression profiles (Alternative-M2, Classical-M1, Hypoxia, Dendritic Cells (DC), Microglial, Mitotic, Neutrophil, and Unknown M). (B) Heatmap of the top gene signatures identified in each myeloid subpopulation. Gene ontologies, from DAVID, enriched in each subpopulation listed. (C) UMAP projections of myeloid subpopulation clusters with pediatric molecular subgroups (PFA1: n = 13, PFA2: n = 7, PFB: n = 1, RELA: n = 5, YAP: n = 1). (D) Proportion of myeloid cells type in each patient sample. Quantification of proportion of myeloid cells within each subpopulation compared across ependymoma molecular subgroup. Samples with at least 50 cells were included in the analysis. Value above each comparison is p value calculated between subgroups. (E) Proportion of myeloid cells of each subpopulation identified in each molecular subgroup in scRNAseq analysis. (F) Proportion of myeloid cells of each subpopulation infiltrating PFA1 versus PFA2 ependymoma in scRNAseq analysis.

The immune microenvironment in ependymoma has historically been inferred through gene signatures identified in bulk transcriptome datasets.^9^^,^^14^ Using a single-cell approach to this problem, we identified 13 transcriptionally unique immune cell subpopulations (Figure S3). These clusters include 8 of myelocytic lineage, 3 of lymphocytic lineage (2 T cell and one B cell), and two unidentifiable clusters (Figure S3). In this study, we focused on myeloid lineages (Figure 1A) as previous studies have suggested a role for the PFA myeloid immune response.^12^ We hypothesized that targeting myeloid cell interactions will be key to relieving microenvironmental immunosuppressive effects on T cells in PFA tumors.

Using the top 50 genes associated with these myeloid cell subpopulations (Table S2), we classified them as alternative-M2, classical-M1, hypoxia, dendritic cells, microglial, mitotic, neutrophil, and undefined-M (Figure 1A). Similar to our previous findings with neoplastic cells,^17^ all five ependymoma subgroups (PFA1, PFA2, PFB, ZFTA-fused, and ST-YAP) contained variable proportions of each subpopulation (Figures 1C and 1D). Gene ontologies from DAVID were used to further characterize each subpopulation (Table S5). Characterizing tumor-infiltrating immune cells in reference to peripheral blood immune cell expression patterns is challenging, as gene expression is heavily skewed by tumor microenvironmental factors. Because of this, many of the markers used to define immune cell lineage are broadly expressed across tumor-infiltrating immune cell subpopulations. For example, CD86, commonly used to define macrophages, is highly expressed in both classical-M1 cells and hypoxia myeloid cells (Figure S4A). Similarly, CSF3R, a gene associated with neutrophils, is highly expressed by the population we defined as neutrophils but also highly expressed in classic-M1 and microglial subpopulations (Figure S4B). Therefore, we classified the myeloid cell subpopulations based on their gene expression characteristics rather than using published immune cell atlases from other anatomic sites. Where lineage was difficult to ascertain (Hypoxia and undefined-M), we defined them under the general classification of myeloid cells.

#### Microglial

While this subpopulation expressed similar genes to the classical-M1 subpopulation, these cells were distinguished by *TMEM119* expression, a signature gene expressed in microglial cells. These cells were abundant in PFA1 tumors but had slightly higher infiltration in PFA2 (Figure 1F).

#### Classical-M1

The gene signature most enriched within this subpopulation of myeloid cells was antigen processing and presentation (Table S4). These myeloid cells exhibited high expression of MHC class II molecules and Fc-receptor signaling genes (Table S4). Though not significant, these cells were seen in greater abundance in PFA2.

#### Alternative-M2

These myeloid cells were characterized by top genes *MRC1* (CD206) and *CD163* (Table S4). Both are signature genes of alternatively polarized myeloid cells. These cells were most abundant in ZFTA-fused tumors and predominating within only two patient samples (UPN 1158 and UPN 1329, Figure 1D). In the bulk tumor gene expression dataset (GSE64415), ZFTA-fused tumors had higher *MRC1* expression than either PFA or PFB (Figure S4).

#### Dendritic cells

The genes associated with this subpopulation were those of dendritic cells, with TCR signaling, antigen processing, T cell activation, and response to interferon-gamma as the predominant signatures (Table S4).

#### Hypoxia myeloid

This was the only subpopulation that we identified, by single-cell RNA sequencing (scRNAseq), to have differential infiltration between PFA1 and PFA2 molecular subgroups with a 2-fold increase of hypoxia myeloid cells in PFA1 compared to PFA2 tumors (Figure 1F). These cells expressed genes associated with stress response, response to oxygen, angiogenesis, wound healing, cell migration, and neutrophil activation. A characteristic gene of this subpopulation is TREM1. (Table S4).

#### Neutrophil

While expressing similar genes as the hypoxia subpopulation, this population of cells are clearly neutrophils with high expression of *S100* genes as well as neutrophil gene signatures *LYZ* and *FCN1* (Table S4). PFA1 samples had non-significantly greater infiltration of neutrophils than PFA2. ST-ZFTA-fused on average had the most infiltration but this was due primarily to UPN 1158 and UPN 870 (Figure 1D).

#### Mitotic

This represented a group of highly mitotic cells without a clear lineage distinction (Table S4).

#### Undefined-M

This cluster of cells did not have a clear lineage distinction. The population expressed genes associated with both classical-M1 and hypoxia subpopulations (Table S4) and was evenly associated with all EPN subgroups in the single-cell analysis (Figure 1E).

### Hypoxia myeloid cells are enriched in PFA1 EPN in independent bulk analyses and present in previous spatial transcriptomic analyses

To validate our single-cell findings, we performed Cibersort analyses on publicly available pediatric ependymoma bulk gene expression datasets with associated DNA methylation profiles supporting the diagnosis of PFA, PFB, or ZFTA-fusion-positive EPN (n = 299). This combined dataset contained 199 PFAs, 41 PFBs, and 59 ZFTA-fusion-positive ependymomas. Where PFA subclassification was known, there were 104 PFA1 and 52 PFA2 tumors.

The distribution of each of the scRNAseq-derived immune cell subpopulations was similar across the bulk Cibersort datasets (Table S2). Consistent with single-cell data, there was low expression of markers associated with T lymphocyte subpopulations (Figure S6).

**Figure 2:**
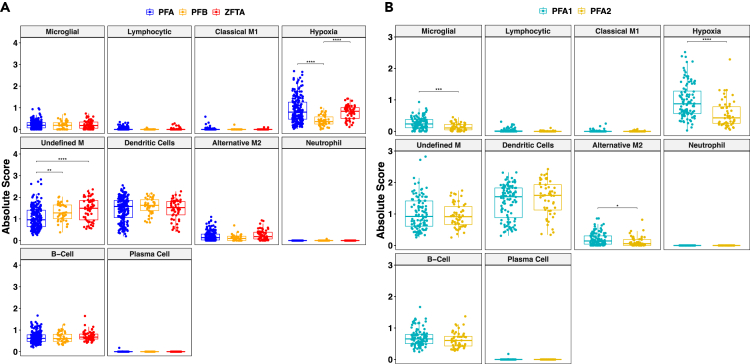
Deconvolution analysis of bulk gene expression validates presence of single-cell myeloid subpopulations (A) ScRNAseq immune subpopulation signature deconvolution (CIBERSORT) was performed on 299 EPN tumor transcriptomes with confirmed molecular subtypes (PFA n = 199, PFB n = 41, ZFTA n = 59)(Wilcoxon test; P-Values with Bonferroni multiple analysis correction. PFA and ZFTA tumors were significantly enriched for the hypoxia myeloid cell subpopulation when compared to the PFB cases (p < 0.0001 in both cases). PFB and ZFTA both were enriched for the Undefined-M signature when compared with PFA tumors (p = 0.016 and p < 0.0001, respectively). No other differences existed after the multiple test correction. (B) Immune subpopulation deconvolution in PFA1 (n = 105) and PFA2 (n = 52). Microglial and alternative M2 group signatures were enriched in PFA1 tumors (p = 0.0004 and p = 0.016, respectively). The hypoxia myeloid subpopulation had the largest magnitude of change with the greatest level of significance between PFA1 and PFA2 tumors (p < 0.0001).

The hypoxia myeloid cell phenotype was significantly overexpressed in ZFTA-fused and PFA tumors compared to PFB (p < 0.0001) (Figure 2A). PFA1 tumors were enriched for both microglial and alternative-M2 subpopulations compared to PFA2 (p = 0.0005 and p = 0.016, respectively). The Cibersort bulk gene expression dataset confirmed the enrichment of the hypoxia myeloid cellular subpopulation in PFA1 compared to PFA2 tumors (p < 0.0001), reflective of the pattern seen in the initial scRNAseq analysis.

In addition to the identification of hypoxia myeloid and classic-M subpopulations in bulk cibersort analyses, these subpopulations were also seen in our recent PFA ependymoma spatial transcriptomic analyses.^19^ When compared to their spatial transcriptomic equivalents, the hypoxia myeloid and classic-M single-cell subpopulations had Jaccard indices of 0.22 and 0.16, respectively. Overlap was also seen between the gene ontologies for these subpopulations.

### Myeloid cell infiltration is intensified close to areas of altered tumor architecture such as necrosis and vasculature

Single-cell analysis by gene expression profiling or flow cytometry usefully identifies unique cellular subtypes not previously detectable with bulk tumor transcriptomics and methylomics. However, single-cell analyses are not spatially informed. Conversely, spatial transcriptomics provides both spatial and gene expression data, but does not obtain single-cell resolution gene expression data.

The previously published spatial transcriptomic analysis provided initial evidence that ependymoma myeloid cells are located close to borders between epithelial and mesenchymal tumor areas and near to areas of tumor necrosis.^19^ Given this restricted distribution of myeloid cells at the gene expression level, we sought to establish whether this was recapitulated at the protein level.

**Figure 3:**
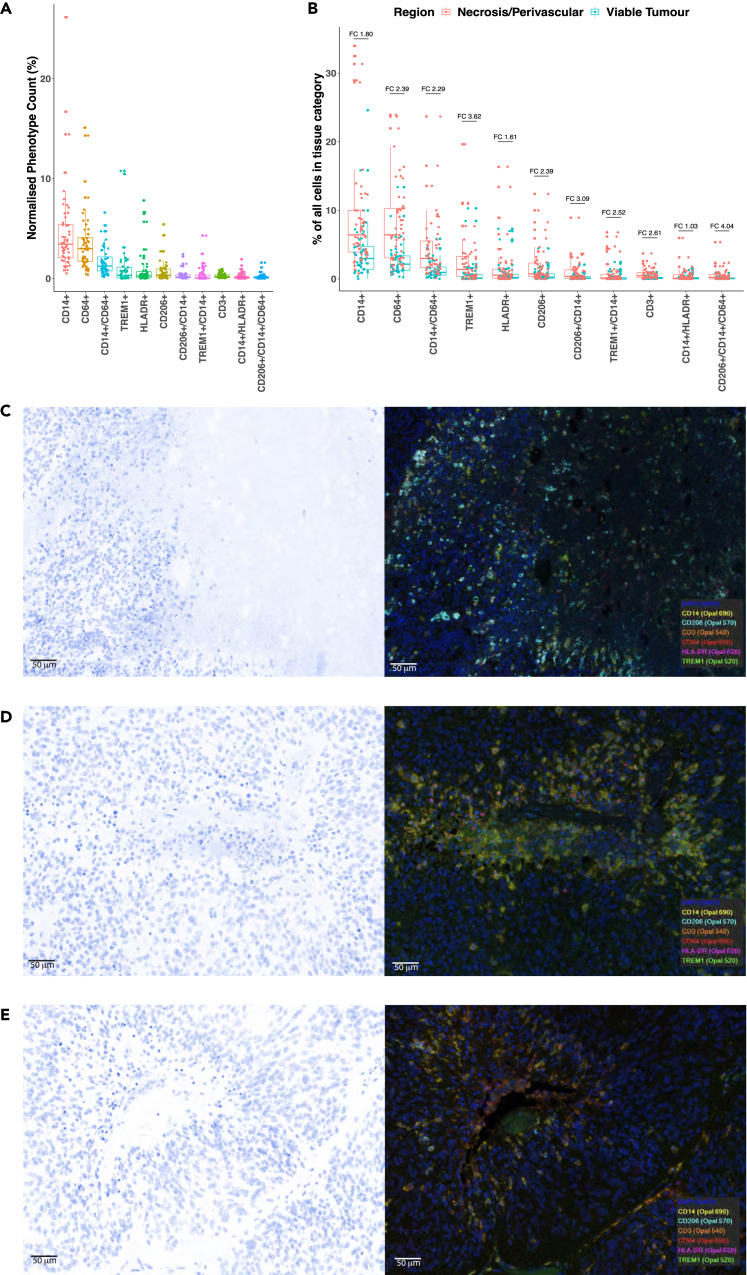
Immune cells corral around necrotic regions and perivascular niches (A and B) each dot is representative of a single cell identified with that phenotype. (A) Myeloid phenotypes normalized by total number of cells indicated that myeloid cells constitute approximately 0.5%–5% of all cell phenotypes in the tumor sections analyzed all though some samples express significantly more myeloid markers. (B) All myeloid panel phenotypes were associated with greater levels of expression in regions of tumor compared with necrotic or perivascular niches (p < 0.0001, chi-square test). In view of the large numbers of cells included in this analysis, the bars for each phenotype indicate the fold change to give an insight into the possible biological significance of each difference. The presence of more immune cells outside of the main tumor parenchyma provides supporting evidence for ependymoma as either an immune desert or immune-excluded type tumor. (C) Area of transition between tumor and necrosis characterized by multiple CD206, CD14, HLADR, and TREM1-positive cells. (D) Area of central necrosis surrounded by a ring of myeloid cells staining positive for CD14, HLADR, and CD64. (E) Blood vessel surrounded by multiple myeloid related cells staining positive for multiple myeloid markers. Note that the tumor beyond the myeloid cell ring is largely devoid of positively staining cells. Images captured using 20X objective and scale bar is 50 μm.

The myeloid multiplex immunofluorescence panel, consisting of CD14, CD64, CD3, CD206, HLADR, and TREM1, was applied across 52 PFA EPN sections. Myeloid cells constituted on average between 0.5% and 10% of all cells (Figure 3A). This proportion correlated well with the 5% of cells estimated to be myeloid cells in the Cibersort signature published by Gillen et al.^17^ The most highly expressed cell marker was CD14 (median 3.43% of cells) followed by CD64 (median 3.00% of cells). CD3^+^ cells were rare and less variably expressed (median 0.19% of all cells, range 0.03%–0.94%), consistent with our gene expression data showing low T lymphocyte infiltration and activity in PFA ependymoma (Figure 3A).

All immunophenotypes were present in higher proportions in necrotic and perivascular niches and at regions of transition between densely packed tumor cells and necrosis, validating, at the protein level, our recent spatial transcriptomic analysis as transitions between mesenchymal and epithelial regions^19^ (Figures 3B–3E). For each immune cell phenotype, epithelial regions contained significantly smaller proportions of myeloid cell markers (p < 0.0001 for each immune cell category). However, the large number of cells analyzed means that small variations in the sizes of cellular populations could result in highly significant p values. Therefore, fold changes between epithelial and necrotic and perivascular areas for each immune cell marker were calculated to better understand the potential biological significance of these changes (Figure 3B). Myeloid phenotypes with the highest fold changes between densely packed tumor cells and necrotic and perivascular areas were CD206^+^/CD14^+^/CD64^+^ (FC.404), CD206^+^/CD14^+^/CD64^-^ (FC3.09), and TREM1^+^ (FC3.62). All phenotypes other than CD14^+^, HLADR^+^, and CD14^+^/HLADR^+^ at least doubled in necrotic and perivascular areas, compared to epithelial tumor areas.

### Hypoxia myeloid cells localize to necrotic and perivascular niches and are positively correlated with MEC tumor cells

At the mRNA level, the myeloid subpopulations identified by our recent spatial transcriptomic analysis located to specific “mesenchymal” regions characterized by the presence of MEC tumor cells.^19^ Through the myeloid multiplex immunofluorescence panel in this study, we have established that myeloid populations are enriched in areas of necrosis and vasculature and at transition points away from epithelial zones. Additionally, based on our single-cell analysis, hypoxia myeloid cells share several genes with the MEC tumor cell subpopulation. We then hypothesized that the hypoxia myeloid cells gene expression profile would therefore correlate with the MEC tumor profile. All four bulk gene expression datasets analyzed using Cibersort, demonstrated a high correlation coefficient linking hypoxia myeloid and MEC subpopulations (Denver, r = 0.92, 95% CI 0.85–0.95, p < 0.0001, Nottingham, r = 0.83, 95% CI 0.72–0.89, p < 0.0001, Heidelberg, r = 0.82, 95% CI 0.76–0.86, p < 0.0001, St. Jude, r = 0.91, 95% CI 0.84–0.95, p < 0.0001) (Figure S7A). Of note, while the hypoxia myeloid gene expression profile correlates with MEC subpopulations, only 11% of the genes are shared between the two subpopulations indicating they are different subpopulations of cells (Table S6; Figure S7B).

**Figure 4:**
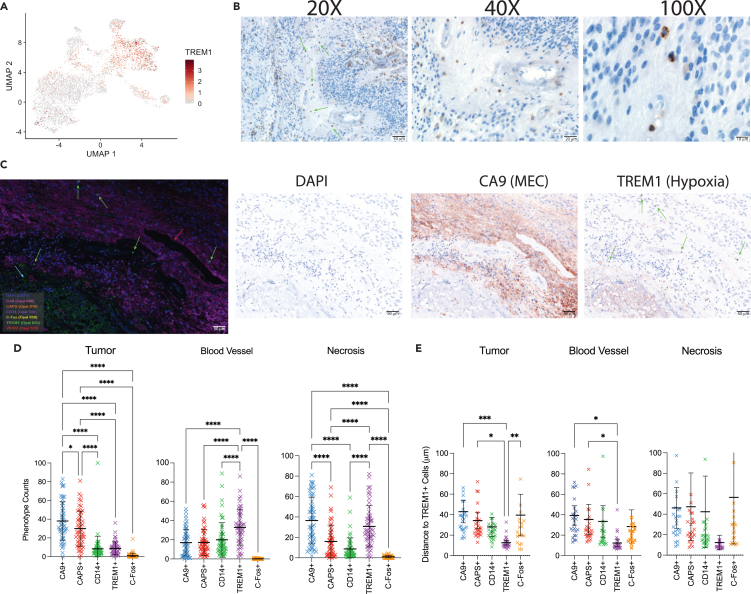
Hypoxia myeloid cells are localized to regions of necrosis (A) *Trem1* gene expression distribution in single-cell RNAseq myeloid subpopulations. (B) Representative images of Trem1 antibody stain on immunohistochemistry FFPE slides of PFA ependymoma. Images captured using 20X, 40X, and 100X objectives and scale bars are denoted 50 μm, 20 μm, and 10 μm, respectively. (C) Representative multi-analyte immunofluorescent images of FFPE PFA samples. Area of necrosis denoted with blue arrow and a blood vessel denoted with red arrow. TREM1+ cells are indicated with green arrows. Pink fluorophore is CA9 antibody staining. Single-stain immunohistochemistry images for the same region. DAPI is nuclear staining, CA9+ are MEC tumor cells, and TREM1+ are hypoxia myeloid cells. Images captured using 20X objective and scale bar is 50 μm. (D) Quantification of cell phenotypes stratified by tissue segmentation on 59 (n = 30 Denver and n = 29 Nottingham) FFPE slides with 5 regions per slides selected for analysis. Mean and 95% confidence interval denoted by bars. ∗∗∗ denotes p value <0.001. ∗ denotes p value <0.05. (E) Nearest neighbor analysis calculating the distance between TREM1+ hypoxia myeloid cells, tumor cells, and other myeloid cells stratified by tissue segmentation. Mean and 95% confidence interval denoted by bars. ∗∗∗ denotes p value <0.001. ∗ denotes p value <0.05.

Given the correlation of gene expression by Cibersort and spatial transcriptomics, we hypothesized that the hypoxia myeloid cells would also colocalize at the protein level with MEC tumor cells. We used TREM1 to identify the hypoxia myeloid population. *TREM1* is one of the top enriched genes identified in the hypoxia subpopulation with 60.3% of hypoxia myeloid cell expression and only 15% of all other cell expression (logFC 0.768, p value <0.001, pct_in 60.28, pct_out 14.94) (Figure 4A). Furthermore, *TREM1* expression was rarely seen in EPN neoplastic subpopulations (https://www.pneuroonccellatlas.org).

The TREM1-positive cells have an ambiguous cytomorphology reminiscent of a monocyte with modest cytoplasm and a mono-lobated nucleus. Immunohistocompatibility (IHC) also showed that TREM1+ myeloid cells are largely localized to the interface of necrosis and viable tissue, most frequently in a perivascular and intravascular distribution (Table S7; Figure 4B). This finding suggests that the TREM1+ cells may be associated with the MEC tumor population, which we have previously described as being enriched in PFA1 tumors and localized to perinecrotic zones. This is supported by parallel IHC analysis of subpopulation-specific markers in the same cohort of PFA EPN which showed the highest TREM1 correlation was with CA9, a marker of MEC (r2 = 0.92, p < 0.001, n = 49)(Figure S7).

Given the correlation between TREM1+ cells and MEC subpopulation marker, we performed multi-analyte immunofluorescent imaging of FFPE slides to examine cellular spatial relationships. We used previously described EPN tumor subpopulation markers, CA9 (MEC), CAPS (CEC), Fos (UEC), and VIPR2 (TEC), along with TREM1 and DAPI. We scanned 5 histologically distinct regions for each patient sample (Denver cohort n = 30, Nottingham cohort n = 24) on the whole slide image and segmented each region into tumor, necrosis, or blood vessel. The training algorithm gave a 93% accuracy for identifying tissue segmentation in the 15-image training set. For the tissue segmentation, we aligned each region with the corresponding H&E region and a pathologist (N.W.) identified each feature within that region (Figure S1). VIPR2 was excluded from the cell phenotype analysis as it was ubiquitously stained throughout the slide such that it was difficult to identify true staining. The antibodies selected for this panel are specific for each cell type and therefore cell phenotyping was performed on the single antibody-positive cells (Figure S1). Consistent with our IHC results, TREM1+ cells were enriched in necrosis and blood vessel regions (30.8% and 32.9%, respectively) (Figures 4C and 4D). CA9+ MEC cells were the most abundant cell population in the necrosis regions (36.6%) and were also enriched in the tumor regions on the borders of necrosis. Most of the CA9+ MEC cells were within 50 μm of TREM1+ cells in the necrosis regions (Figure 4E). Additionally, there was an increase in CD14^+^ myeloid cells in the blood vessel regions. Unsurprisingly, CAPS+ cells were enriched in epithelial regions of tumor (29.9%). Interestingly, the CAPS+, CEC, cells were similar distances from the TREM1+ cells as were the MEC cells suggesting the hypoxia myeloid cells may facilitate transforming the CEC cells to MEC phenotype. C-Fos+ cells were sparse and equally distributed between the different spatial regions, consistent with the role of UEC as a progenitor cell of both epithelial and mesenchymal regions in our spatial transcriptomic study.^19^

### Hypoxia myeloid cells have an immune-suppressive phenotype

We have previously shown that PFA1 EPN harbors an immune-suppressive phenotype which we hypothesize leads to multiple recurrences and poor overall survival.^9^ Given the hypoxia myeloid cells are enriched in PFA1 tumors, we next focused on describing these cells in greater detail. In our prior bulk microarray analysis, we showed enrichment of the IL-6/STAT3 pathway in EPN tumor flow-sorted myeloid cells.^12^ To determine whether this held in the scRNAseq, we performed a regulatory network inference analysis that infers the transcription factor activity at the single-cell level. Consistent with our prior findings, STAT3 was among the top transcription factor pathways upregulated in the hypoxia myeloid compartment (Table S8). We also found that retinoic acid receptor signaling was upregulated. This is of interest as we found the tretinoins to be highly effective at promoting PFA1 tumor cell death *in vitro* and are actively pursuing this class of chemotherapy agents as a potential maintenance regimen for high-risk EPN patients.^20^

### Hypoxia myeloid cells secrete immune-suppressive cytokine IL-8

We have previously reported the significance of cytokine signaling in PFA EPN immunobiology. Tumor-secreted interleukin-6 (IL-6) induces STAT3 signaling in infiltrating monocytes which results in secretion of IL-8.^12^ Furthermore, the IL-8-producing myeloid cells can further amplify the immune-suppressive response by polarizing naive monocytes to a pro-tumor phenotype. We therefore hypothesized that hypoxia myeloid cells are one of the myeloid subpopulations involved in this process.

**Figure 5:**
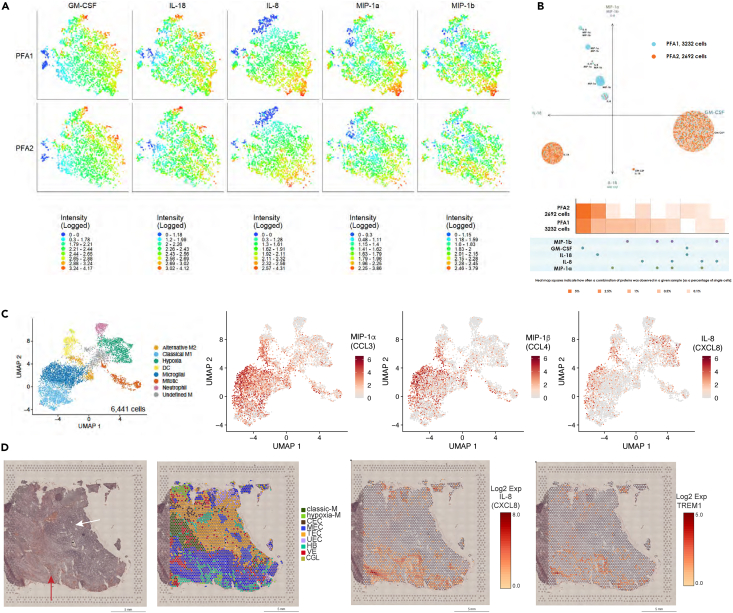
Functional phenotyping of single-cell subpopulations Monocytes were isolated from 6 PFA1 and 5 PFA2 samples using magnetic bead separation. (A) 2D t-SNE (2D t-distributed stochastic neighbor embedding) analysis indicates greater signal intensity of multiple cytokines co-secreted by PFA1 monocytes than PFA2 monocytes. (B) PAT-PCA (polyfunctional activation topology-principal component analysis) shows that 9 subpopulations of monocytes were identified based on polyfunctional cytokine secretion of MIP-1α, MIP-1β, GM-CSF, IL-18, and IL-8. PFA1 exhibits higher polyfunctional heterogeneity at single-cell level as PFA2 mainly secretes GM-CSF or IL-18. Density and size of circle indicate number of cells in each subpopulation. Heatmaps compare the percentage of single cells secreting various monofunctional and polyfunctional groups across multiple samples. Secretions in PFA1 display as both more polyfunctional and with higher secretion frequency than PFA2. For example, the combined secretions of MIP-1α and MIP-1β, and MIP-1β and IL-8 are unique to PFA1 compared to PFA2. (C) Single-cell RNAseq UMAPs of *CCL3* (MIP-1α), *CCL4* (MIP-1β), and *CXCL8* (IL-8) gene expression of ependymoma tumor-infiltrating myeloid cells. *CCL3* (MIP-1α) expression is enriched in microglia, M1 and some M2 myeloid cells. *CCL4* (MIP-1β) gene expression is localized to a portion of microglial cells. *CXCL8* is enriched in hypoxia myeloid cells and *CCL4* expression microglial cells. (D) Representative spatial transcriptomics slides. Left: H&E stain of PFA1 ependymoma tumor sample. Red arrow denotes region of necrosis and white arrow denotes a region of hypercellularity with prominent perivascular pseudorosettes. Middle Left: H&E image overlayed with spatial transcriptomics spot clusters. Middle Right: Spatial gene expression of *CXCL8* (IL-8). Right: Spatial gene expression of *TREM1*. Scale bar: 5 mm.

To determine the cytokine profiles of infiltrating EPN myeloid cells, we utilized the IsoLight and IsoSpark platforms that can measure cytokine and chemokines secretion at the single-cell level. We isolated CD45^+^ cells from PFA single-cell suspensions (6 PFA1, 5 PFA2) using magnetic bead isolation. CD45^+^ cells were incubated with lipopolysaccharide for 24 h to enhance immune functional characteristics, and then loaded onto the Innate Immune Single-cell Secretome chip (IsoPlexis). Similar to scRNAseq data, we detected different subpopulations of cells based on cytokine/chemokine secretion profiles. Samples were pooled based on methylation phenotype and analysis results were clustered based on the polyfunctional strength index (Figure 5A), annotated from IsoSpeak software. PFA1 samples were enriched for a distinct cluster of IL-8-producing cells and an additional cluster secreting IL-8 in combination with macrophage inflammatory protein-1 alpha (MIP-1α) and MIP-1β (Figures 5A and 5B). To make certain these cytokine release profiles were not dependent of LPS stimulation, we validated these findings in a multiplex cytokine release assay using media supernatant collected from unstimulated CD45^+^CD11b+ myeloid cells flow sorted from single-cell suspensions (Figure S8A). This experiment showed that cytokine secretion observed by the LPS-stimulated single-cell data is not an LPS-mediated response.

Interestingly, samples that had high infiltration of hypoxia myeloid cells by scRNAseq had subsets of myeloid cells secreting MIP-1β, MIP-1α, and IL-8 either individually or in combination (Figure 5C). MIP-1α and MIP-1β are both chemoattractants and are encoded by *CCL3* and *CCL4* genes. Both genes are significantly enriched in a subset of the microglial subpopulation, being detected in 81% and 54% of cells, respectively (p < 0.001). *CCL3* was also enriched in the M1 subpopulation with 60% of cells expressing CCL3 (log FC 0.64, p < 0.001) (Figure 5C). Additionally, *CCL3 and CCL4* were highly enriched in the chemotactic-M spot clusters identified in our prior spatial transcriptomics publication.^19^ These chemotactic-M spots were located along the borders of necrosis where epithelial-to-mesenchymal transition was occurring.

IL-8 (*CXCL8*) was found in 42.5% of hypoxia myeloid subpopulation and 31.4% of the microglial subpopulation (p < 0.001). Evaluating the spatial orientation of IL-8 expression within the tumor microenvironment, we utilized the spatial transcriptomics dataset from our previous publication.^19^ IL-8 (*CXCL8*) gene expression was enriched in the hypoxia myeloid subpopulation and MEC-D spots (Figure 5D). MEC-D is denoted as being mesenchymal ependymal tumor cells; however, when we overlayed *TREM1* expression, we found most of the gene expression located in the MEC spot clusters with similar expression pattern as IL-8, suggesting intermixing of hypoxia_M with MEC-D cells. This is a limitation of spatial transcriptomics; the capture spots are 50 μm in diameter and contain 20–30 cells. Nearest neighbor analysis from spatial proteomics found the MEC cells (CA9+) were an average of 45 μm from TREM1+ cells (Figure 4E). This suggests the IL-8 and TREM1 gene expression seen in the MEC spots is from the proximity of hypoxia myeloid cells to MEC tumor cells in the regions of necrosis. Collectively, spatial proteomics and transcriptomics lead to the hypothesis that microglial cells are responding to tumor necrosis by recruiting additional myeloid cells to the regions between densely packed tumor cells and necrosis.

In PFA tumors with low hypoxia myeloid infiltration, cytokine profiles were more antitumor, characterized by higher production of GM-CSF and IL-18 (Figure 5A). These cells are functionally distinct from the IL-8, MIP-1α, and MIP-1β-producing cells and are enriched in PFA2 (Figure 5B). *IL18* gene expression was enriched in the microglial and M1 subpopulations (Figure 5B) which are also both slightly higher in PFA2.

Taken together with our previous findings in PFA immunobiology, these data provide evidence that hypoxia myeloid cells significantly contribute to the generation of the EPN immune-suppressive environment. Our studies on the PFA ependymoma myeloid environment give a new insight into key tumor and immune interactions and a hypothetical mechanism of immune evasion in PFA.

## Discussion

Enhanced understanding of the biology of posterior fossa EPN is critical for the development of novel rational treatments. We utilized scRNAseq and spatial proteomics to generate deeper insight into the tumor immune microenvironment (TME). We have identified eight transcriptionally discrete subpopulations of infiltrating myeloid cells and have described the spatial distribution of myeloid cells in PFA ependymoma. A subpopulation of hypoxia myeloid cells enriched in PFA1, with characteristics of MDSC, was associated with an immune suppressive TME. We hypothesize that this immune suppressive environment must be a key target for the development of new therapies for EPN, which may also increase the potential efficacy of more conventional approaches such as chemotherapy that may not have had their full therapeutic potential optimized.^21^^,^^22^^,^^23^^,^^24^^,^^25^^,^^26^

Previous work from our group^17^ and others^18^ identified multiple unique neoplastic EPN subpopulations. Of these, MEC were most strongly associated with aggressive PFA1 tumors. We hypothesized a pro-tumor, immunosuppressive link between MEC and hypoxia myeloid cells within the TME. This hypothesis is supported by the strong correlation between MEC and hypoxia myeloid phenotypes across four independent ependymoma gene expression cohorts, spatial proteomic analyses using both single-stain immunohistochemistry and multiplex immunofluorescence in our study, alongside previously reported spatial transcriptomic data.^19^ Further *in vitro* and *in vivo* studies are now required to test these findings in the process of translation to the clinic.

Previous studies of PF EPN have shown the importance of tumor-infiltrating immune cells and associated ontologies in predicting outcomes and establishing a role for differential immune function across molecular subtypes.^9^^,^^12^^,^^13^^,^^14^^,^^16^^,^^27^^,^^28^ Our single-cell approach improves the resolution of the previous gene expression findings. We previously showed the same cellular subtypes appear across all molecular entities of EPN and assignment to specific molecular subgroups is directly related to the proportion of cellular subpopulations in the entire tumor.^17^ Our study is consistent with these findings, showing both PFA subgroups exhibit all the myeloid subpopulations in variable proportions. The infiltration of the hypoxia myeloid cells provides an explanation for the immunobiology previously identified in PFA1.^9^^,^^17^ Our findings are consistent with a scRNAseq study on adult spinal EPN that identified high levels of intratumor heterogeneity and highlighted the importance of tumor-associated macrophages, particularly related to driving inflammation and angiogenesis,^29^ both functions strongly associated with PFA EPN.^3^^,^^9^^,^^27^^,^^28^

Hypoxia myeloid cells exhibit an MDSC phenotype with features including activation of STAT3 pathway, likely in response to tumor-secreted IL-6. Hypoxia-M cells also have a strong IL-8 (*CXCL8*) gene signature which provides support for the hypothesis that IL-8-secreting cells, identified using single-cell cytokine release assays, are hypoxia myeloid cells. We previously described that IL-8 was the only cytokine significantly upregulated in monocytes cultured in PFA1 conditioned media and could be attenuated by blocking IL-6. Furthermore, the IL-8 secretion, from polarized monocytes, downregulated HLA-DR and CD64 while upregulating immune-suppressive cytokines.^12^ These data would suggest the polarized monocytes are the hypoxia myeloid cells and further studies are needed for validation of this theory.

Interestingly, *IL6* gene expression is exclusively enriched in the MEC subpopulation, suggesting IL-6 is facilitating the transition of the infiltrating myeloid cells to the hypoxia phenotype. This hypothesis is consistent with known patterns of monocytic-MDSC recruitment and development where expansion of the MDSC population is achieved through chronic inflammation triggering with STAT3 responses followed by activation of recruited cells via inflammatory cytokines including IL-6.^30^ The transcriptional profile of the hypoxia myeloid cells is consistent with this explanation, as is the corralling of the immune cells away from epithelial tumor regions into the mesenchymal zones seen in our proteomic analyses. Our spatial profiling confirms that the hypoxia myeloid cells co-localize with MEC in regions of necrosis and perivascular niches.^19^ Hypoxia is critical for the maintenance of PFA EPN, likely because of its developmental origins.^31^ However, our study is the first to propose a mechanistic link between hypoxia and development of an immunosuppressive TME driven through areas of necrosis. While the hypoxia myeloid cells are critical cellular components of PFA1 EPN, other myeloid phenotypes may exert other functions as they corral around necrotic and vascular areas and this is an area requiring more detailed investigation.

We have identified a process in PFA EPN, which, if targeted through therapeutic modulation of the immune environment, may potentiate the efficacy of promising cellular therapies such as CAR-T cells.^32^ These cellular therapies are urgently needed to deliver better outcomes for children with this devastating disease but, without designs incorporating detailed knowledge of the underlying immunobiologic processes, are at risk of failure. Children with particularly high-risk ependymoma, for example those with tumors harboring chromosome 1q gain and 6q loss for which conventional therapies will provide little benefit, need to be considered as early candidates for new agents based on our evolving biological knowledge.^33^ Considering this study, and our prior work, putative agents include the IL-6 receptor antagonist tocilizumab, IL-8 antagonists, and all-trans retinoic acid.

The interactions we have proposed between hypoxia myeloid cells and MECs provides both a rational pathway to target and a route for further investigation. Addressing the immunosuppressive environment generated by tumor-hypoxia-myeloid cell interactions will contribute to better immunotherapeutic approaches and may also serve as an adjunct to potentiate the efficacy of current standard-of-care treatments.^34^

### Limitations of the study

This is study is largely descriptive using a multi-omics approach to characterize ependymoma-infiltrating myeloid cells. This study generated new hypotheses regarding the cellular interactions and cellular function within the complex tumor microenvironment. *In vitro* and *in vivo* experiments modeling these cell-cell interactions are necessary and will be the subject of future studies. The mechanism driving the development of hypoxia myeloid cells remains to be determined although prior work would suggest that it is through mesenchymal EPN tumor cell secretion of IL-6 and hypoxic conditions within the regions of necrosis. Future *in vitro* co-culture studies will be needed to validate this mechanism.

## STAR★Methods

### Key resources table

**Table:** 

REAGENT or RESOURCE	SOURCE	IDENTIFIER
**Antibodies**		

TREM1	Abcam	ab225861; RRID NA
CA9 rabbit Pab	Novus	NB100417; RRID:AB_10003398
CAPS rabbit Pab	Novus	NBP1-91746
c-Fos	Origene	TA806833; RRID: AB_2628246
VIPR2 (VPAC2)	Thermo Fisher	PF3-114; RRID:AB_2216680
HLA-DR	Abcam	Ab20181; RRID AB_445401
CD206	Novus	NBP2-52927; RRID NA
CD64	Abcam	Ab140779; RRID NA
CD3	Leica	PA0553; RRID NA
CD14	Abcam	Ab183322; RRID AB_2909463
DAPI	Akoya	FP1490; RRID NA
HRP-conjugated secondary polymer	Akoya	AHR1001Ea; RRID NA
Opal 520 Reagent Pack	Akoya	FP1487001KT; RRID NA
Opal 540 Reagent Pack	Akoya	FP1494001KT; RRID NA
Opal 570 Reagent Pack	Akoya	FP1488001KT; RRID NA
Opal 620 Reagent Pack	Akoya	FP1495001KT; RRID NA
Opal 650 Reagent Pack	Akoya	FP1496001KT; RRID NA
Opal 690 Reagent Pack	Akoya	FP1497001KT; RRID NA

**Biological Samples**		

Human surgical samples	University of Colorado/Morgan Adams Foundation Tumor Bank	Refer to Table S1
Human surgical samples	University of Nottingham	Refer to Table S1

**Critical commercial assays**		

Allprep RNA/DNA Mini Kit	QIAGEN	80204
Chromimum Single cell V2 and V3 Chemistry	10x Genomics	120237
Library Kit, Gel Bead and Multiplex Kit	10x Genomics	120262
Chip Kit	10x Genomics	120236
Single-Cell Innate Immune Chip	Isoplexis	ISOCODE-3L02-44
CD45^+^ MicroBeads, human	Miltenyi Biotec	130-188-780

**Deposited data**		

scRNAseq and Affymetric microarray data	This Study	GSE125969
DNA methylation profile data	This Study	GSE190798
EPN immune scRNAseq browsable web resource	This Study	pneuroonccellatlas.org
Expression data from human ependymoma	Hoffman et al.^9^	GSE50385
Gene expression data from ependymal tumor samples	Pajtler et al.^3^	GSE64415
Gene expression data from posterior fossa ependymomas	Pajtler et al.^7^	GSE100240

**Software and algorithms**		

Bioconductor R (version 3.5.3)	Roswell Park Comprehensive Cancer Center	https://www.bioconductor.org
CellRanger (version 2.1.1)	10x Genomic	https://support.10xgenomics.com/single-cell-gene-expression/software/overview/welcome
Seurat (version 4.0.3)	Satija laboratory^35^	https://cran.r-project.org/web/packages/Seurat/
gProfiler2 (version 0.2.0)	Kolberg et al.^36^	https://cran.r-project.org/web/packages/gprofiler2/
pySCENIC (version 0.9.18)	Van de Sande B et al.^37^	https://github.com/aertslab/pySCENIC
DAVID (version 6.8)	NCI	https://david.ncifcrf.gov/
Harmony (version 0.1.0)	Korsunsky et al.^38^	https://www.nuget.org/packages/Harmony/0.1.0
Presto (version 1.0.0)	Korsunsky et al.^39^	https://github.com/immunogenomics/presto
MolecularNeuropathology.org version 12	German Cancer Research Center (DKFZ)	https://www.molecularneuropathology.org/mnp
gSNAP	Wu and Nacu^40^	N/A
Cufflinks	Trapnell et al.^41^	N/A
IngerCNV	Broad Institute	https://github.com/broadinstitute/inferCNV
IsoSpeak	Isoplexis	https://phenomex.com/products/isospeak-software/
InForm Tissue Analysis Software (version 2.5.1)	Akoya Biosciences	https://www.akoyabio.com/phenoptics/software/inform-tissue-finder/
Phenochart Whole Slide Viewer (version 1.1.0)	Akoya Biosciences	https://www.akoyabio.com/support/software/
Phenochart Whole Slide Viewer (version 1.1.0)	Akoya Biosciences	https://www.akoyabio.com/support/software/
Vectra 3 (version 3.0.7)	Akoya Biosciences	https://www.akoyabio.com/phenoptics/mantra-vectra-instruments/vectra-3-0/
phenoptrReports	Akoya Biosciences	https://www.akoyabio.com/phenoptics/software/phenoptrreports/
Illustrator 23.0.3	Adobe	N/A
Prism 9	Graphpad	N/A

### Resource availability

#### Lead contact

Requests for further information and reagents may be directed to and will be fulfilled by the Lead Contact, Timothy Ritzmann (timothy.ritzmann@nhs.net).

#### Materials availability

This study did not generate any new unique reagents.

### Experimental model and study participants details

Surgical material was collected and processed at Children’s Hospital Colorado as previously described^15^(COMIRB 95–500). Clinical data for each patient, including ependymoma molecular subtype can be found in Table S1. Single-cell RNAseq (n = 27) and single-cell cytokine analyses (n = 12) were performed. Sequential slices from archived paraffin-embedded samples were used for immunohistochemistry (n = 45) and immunofluorescence (n = 27) (Table S1).

Formalin-fixed, and paraffin embedded (FFPE) samples were used for whole transcriptome RNAseq, immunohistochemistry, and multiplex immunofluorescence (Table S1). All UK samples were handled and monitored in accordance with the Human Tissue Act (UK) and ethical approval was in place for use (Research Ethics Committee (UK) ref. 05/MRE04/70).

Ethnicity is reported for the Denver patient cases. During the collation of the Nottingham/UK archival cohort, ethnicity status was not routinely collected and therefore is not available for reporting here. None of our analyses were designed to consider patient ethnicity. We are not aware of any evidence of differences in microenvironmental constitution in pediatric ependymoma that relates to ethnic origin in the current literature.

### Method details

#### ScRNAseq methods

To maximize the use of our banked single-cell suspensions, we reanalyzed the same single cell capture dataset reported in our previous manuscript.^17^ Briefly, viably frozen single-cell suspensions were thawed in batches and live cells were isolated using propidium iodide exclusion. We sequenced a minimum of 2000 cells per sample, utilizing the Chromium Single Cell V2 and V3 Chemistry Library Kits, Gel Bead & Multiplex Kit and Chip Kit (10X Genomics). RNA transcripts were converted to cDNA, barcoded and sequenced on Illumina HiSeq4000 and Nova-Seq6000 sequencers to obtain approximately 50 thousand reads per cell.

#### ScRNAseq data analysis

Whole tumor EPN single cell RNA-seq data were obtained from GSE125969. Myeloid and lymphocyte populations were extracted from the whole tumor dataset, using published annotations from our previous manuscript.^17^ The Seurat single cell toolkit was used to perform normalization, clustering, and to generate Uniform Manifold Approximation and Projection (UMAP) visualizations.^35^ Harmony integration (theta = 1) was performed to correct batch effects between individual patient samples using 20 dimensions for the combined lymphocyte and myeloid dataset.^38^ We did not perform doublet detection and removal on these samples due to concern with the utility and accuracy of these algorithms on our data. The expected doublet rate for these samples is low (1–2%) because we captured generally fewer than 2,000 cells per sample. We also do not observe rare cell populations with mixed cell type markers which could be indicative of doublets, suggesting that doublet removal is unlikely to change the conclusions of our study. Harmony embeddings were then used as input for clustering and generation of UMAP visualizations. T cells were extracted from the combined dataset, normalized using scTransform, and integrated with Harmony using 10 dimensions to identify additional T cell subpopulations. Marker genes of subpopulations were identified by comparing cells in each subpopulation to all other cells in each tested comparison using wilcoxon rank sum tests from the Presto package. Enriched GO-terms were identified using gProfiler2 by selecting the top 500 marker genes for each subpopulation. Single-cell regulatory network inference and clustering (pySCENIC) was used to infer transcription factor activity at the single cell level.^37^

A browsable web interface for this EPN immune scRNA-seq data is available at the Pediatric Neuro-oncology Cell Atlas (http://pneuroonccellatlas.org), allowing users to study the cellular restriction and expression level of transcripts of interest at the single-cell level.

#### Bulk RNA-Seq from FFPE tissue (Nottingham cohort)

RNA was extracted from 106 FFPE ependymomas using the FFPE Allprep kit (Qiagen, Germany). Sequencing was performed by Exiqon (Denmark) using the Illumina TruSeq Stranded Total RNA with RiboZero Gold kit. 600 ng RNA was ribodepleted for each sample. Ribodepleted RNA underwent enzymatic fragmentation. First and second strand synthesis was performed before purification of double stranded cDNA. cDNA was end repaired and 3′ adenylated before ligation of Illumina sequencing adaptors. Stranded libraries were amplified using PCR and purified. Size distribution of the libraries was validated on a Bioanalyzer. Libraries were quantified, normalized and pooled before requantification with qPCR. Clusters were generated on the flow cell surface using the optimal pool library concentration before sequencing on the Illumina HiSeq2500 using v4 reagent kits. 100 base pair, paired-end RNA-seq targeting 50 million reads per sample was performed. Fastq files underwent trimming using trimmomatic^42^ before filtering of abundant sequences (rRNA). Remaining reads were aligned to Hg19 and the transcriptome (Gencode GrCh37 Version 11) using TopHat2.^43^ Reads were counted in FeatureCounts^44^ and transformed for visualization using the DeSeq2 R-log transformation.^45^ To identify poorly performing samples the data underwent unsupervised hierarchical clustering which identified a group of 21 failed samples with a low proportion of aligned reads. The remaining cases generated a median of 35,000,000 reads per sample and clustered correctly by tumor location (posterior fossa or supratentorial). The only cases included in this study are the 54 samples which (1) were not in the failed sample cluster, (2) clustered in the correct anatomical location and (3) had a matched DNA methylation profiling result indicating the molecular subgroup.

Whilst historically fresh frozen samples have been preferred for RNA-seq, recent studies have shown that RNA-seq from FFPE can produce robust sequencing data.^46^^,^^47^^,^^48^^,^^49^ The samples included had a median of 81% aligned reads and use was limited to the validation of the single-cell sequencing findings.

#### Deconvolution

Bulk tumor tissue analyzed via Affymetrix array (Colorado (n = 46) – GSE50385, Heidelberg (n = 160) – GSE64415, St. Jude’s (n = 39) – GSE100240) and RNA sequencing (Nottingham (n = 54) – was imputed into Cibersort to further explore the single-cell sequencing findings.^50^ This combined dataset contained 199 PFAs, 41 PFBs, and 59 ZFTA-fusion positive EPNs. Where PFA subclassification was known, there were 104 PFA1 and 52 PFA2 tumors. A Cibersort signature file for the ependymoma immune subpopulations was generated based on the most highly expressed genes in each single-cell myeloid subpopulation (Table S2). P-values for the bulk gene expression datasets and the single-cell derived signature file had a median of 0 (Range 0–0.4). We also used the signature file generated in our prior publication^17^ to analyze the correlation between tumor and myeloid subpopulations. Cibersort analyses were performed in R on absolute mode with 100 permutations using normalized, but not log converted, counts (Mas5.0 normalization for expression array, TPM normalization for RNA-seq). Additional analyses of gene expression were conducted using R2 (http://R2.amc.nl) on GSE64415 for comparisons across PFA/PFB/ZFTA and on GSE64415, GSE50385 and GSE100240 for PFA1/PFA2 comparisons.

#### DNA methylation profiling

DNA was extracted from paraffin embedded samples using the AllPrep FFPE DNA/RNA extraction kit (Qiagen). DNA methylation profiles were generated using Infinium HumanMethylation450 BeadChip arrays (Illumina) with ependymoma subgroups assigned using the Heidelberg Brain Tumor Classifier (v11.4 and 12.3). Newly generated profiles are deposited at GSE190798.

#### Immunohistochemistry

Immunohistochemistry (n = 43) was performed on 5 mm slices of formalin fixed, paraffin-embedded tumor sections and counter stained with hematoxylin. Neuropathological analysis was completed in a blinded scoring by (N.W, S.P.). All antibody details can be found in Table S3. Initial scoring was performed on the degree of necrosis identified. TREM1 score was quantified based on the relative degree of necrosis with 1 being rare and 4 being abundant. An independent set of 29 DNA methylation supported PFA tumors from the UK were also independently stained with the TREM1 antibody and reviewed.

#### Multispectral fluorescence immunohistochemistry

Tissue was fixed in formalin and paraffin-embedded for multiparameter fluorescence immunohistochemistry. Four mm sections mounted on glass slides were sequentially stained for Panel VHuP115: c-fos, TREM1, CA9, CAPS, VIPR2, CD14, Dapi (Akoya). Panel VHuP116: CD64, CD3, HLADR, TREM1, CD206, CD14, Dapi (Akoya) (Table S3). All antibodies were diluted in Biocare antibody dilutant, except the RTU antibodies. Slides were dewaxed (Leica), heat treated in ER2 or ER1 antigen retrieval buffer depending on the antibody for 20 min at 93°C (Leica), blocked in Antibody (Ab) Diluent (Akoya Biosciences), incubated for 30 min with the primary Ab, 10 min with horseradish peroxidase (HRP)-conjugated secondary polymer (anti-rabbit and anti-mouse, Akoya Biosciences), and 10 min with HRP-reactive OPAL fluorescent reagents (Akoya Biosciences). Slides were washed between staining steps with Bond Wash (Leica) and stripped between each round of staining with heat treatment in antigen retrieval buffer. After the final heat treatment in antigen retrieval buffer, the slides were stained with spectral DAPI (Akoya Biosciences), and cover slipped with Prolong Diamond mounting media (Thermo Fisher). Single stain controls, fluorescence-minus-one controls, and appropriate positive and negative control tissues were used throughout the staining process.

#### Multispectral immunofluorescence imaging

Whole slide scans were imaged on the Vectra Polaris Automated Quantitative Pathology Imaging System (Akoya) using the 20x objective. The images were analyzed with inForm software (v2.4.8, Akoya) to unmix adjacent fluorochromes, subtract autofluorescence, segment the tissue into tumor, vascular and necrotic regions, segment the cells into nuclear compartments, and to phenotype the cells according to morphology and cell marker expression. For the myeloid panel independent projects were created to phenotype each cellular marker, then merged, consolidated, and analyzed in R Studio using Phenoptr Reports (Akoya Biosciences).

#### Quantification of multispectral immunofluorescent imaging

To prepare a training set, images were selected to include 5–6 multispectral images (MSI) per sample. The measured spectra references were checked to be brighter than tissue to ensure an acceptable signal-to-noise ratio. All the signals were multispectrally unmixed (Figures S1 and S2). To remove autofluorescence from the analyzed spectra, a representative autofluorescence slide was included to separate autofluorescence into its own channel. The training images were normalized for exposure and background corrected.

To segment tissue, we created categories for each panel (Figures S1 and S2). Examples of the categories were selected and used to train the segmenter. A small pattern scale was used. The percent accuracy of the tissue segmenter reached over 90%. The tissue segmentation was checked throughout the training images. Errors were corrected and the tissue segmenter was retrained. The training images were checked again for correctness. Next, we segmented cells using DAPI as a nuclear reference. We adjusted the splitting sensitivity and nuclear size until we could identify individual cells (Figures S1 and S2).

To train the software to identify specific cell subpopulations, we created phenotypes for each subpopulation of interest (i.e., HLA-DR+/−) and 40–50 cells of each phenotype were selected for training (Figures S1 and S2). Separate training algorithms were created for each marker and algorithms were applied to the batch sample images. Following the application of the batch algorithm the resulting images were checked and any images felt to significantly misrepresent tumor areas were removed from the analysis. The resulting data was consolidated and summarized using PhenoptR in R Studio.^51^ Data was subsequently analyzed as raw cell counts and cell counts normalized by total numbers of cells. Proportions of cells in different tumor areas (necrosis/viable tumor/perivascular regions) were calculated and statistical significance was assessed through Chi-Square testing and calculation of fold changes.

#### Single-cell cytokine assay

Single-cell suspensions were thawed in batches and allowed to recover for 24 h in RPMI supplemented with 10% fetal bovine serum and 1% penicillin/streptavidin (R10), at 37°C and 5% CO_2_. As previously described, myeloid cells account for 99% of CD45^+^ ependymoma tumor infiltrating cells.^15^ To capture a minimum of 50,000 myeloid cells, we used CD45^+^ microbead isolation (Miltenyi) to eliminate tumor cells, which were then stimulated with 10 mg/ml lipopolysaccharide for 24 h. Following LPS stimulation, cells were stained with membrane stain supplied in the Human Innate Immune Secretome kit (isoplexis) according to manufacture protocol. Each sample was loaded on to a separate IsoCode chip supplied in the Human Innate Immune Secretome kit (isoplexis) and run on either the IsoLight or IsoSpark. Data was analyzed using IsoSpeak (Isoplexis) software. Six PFA1 and five PFA2 samples were used.

### Quantification and statistical analysis

Statistical analyses were performed using R bioinformatics and Prism (GraphPad) software. Details of statistical tests performed are included in figure legends. For all tests, statistical significance was defined as p < 0.05.

## Data Availability

• Newly developed data for this study has been included in the key resources table, Figure information and supplemental information. All data reported in this paper will be shared by the lead contact upon request. • This paper does not report original code. • Any additional information required to reanalyze the data reported in this paper is available from the lead contact upon reasonable request.

## Supplemental information

Document S1. Figures S1–S8

Table S1. Study Participant Details of human subject samples used in the study, related to STAR Methods and key resources table

Table S2. Myeloid Immune Gene Signature Cibersort related to STAR Methods, Figures 2 and S5–S7

Table S3. Antibody dilution and antigen retrieval details related to STAR Methods and key resources table

Table S4. Immune cell gene expression profiles used to identify cellular subtypes in Figure 1

Table S5. Gene Expression Ontologies for immune cell subtypes identified in Figure 1

Table S6. Gene expression profiles for MEC tumor cells and Hypoxia myleoid cells, related to Figure 4

Table S7. TREM1 immunohistochemistry scores and pathology interpretation, related to Figure 4

Table S8. Myeloid cells transcription factor activity, related to Figure 4
