# Supplementary material for: Multi-omic approach identifies hypoxic tumor-associated myeloid cells that drive immunobiology of high-risk pediatric ependymoma
Source: iScience. 2023 Aug 9;26(9):107585. doi: 10.1016/j.isci.2023.107585 (PMC10484966; doi:10.1016/j.isci.2023.107585)
Supplement: Document S1. Figures S1–S8 [file mmc1.pdf]

iScience, Volume *26*

Supplemental information

Multi-omic approach identifies hypoxic tumor-associated myeloid cells that drive immunobiology of high-risk pediatric ependymoma

Andrea M. Griesinger, Kent Riemondy, Nithyashri Eswaran, Andrew M. Donson, Nicholas Willard, Eric W. Prince, Simon M.L. Paine, Georgia Bowes, John Rheaume, Rebecca J. Chapman, Judith Ramage, Andrew Jackson, Richard G. Grundy, Nicholas K. Foreman, and Timothy A. Ritzmann

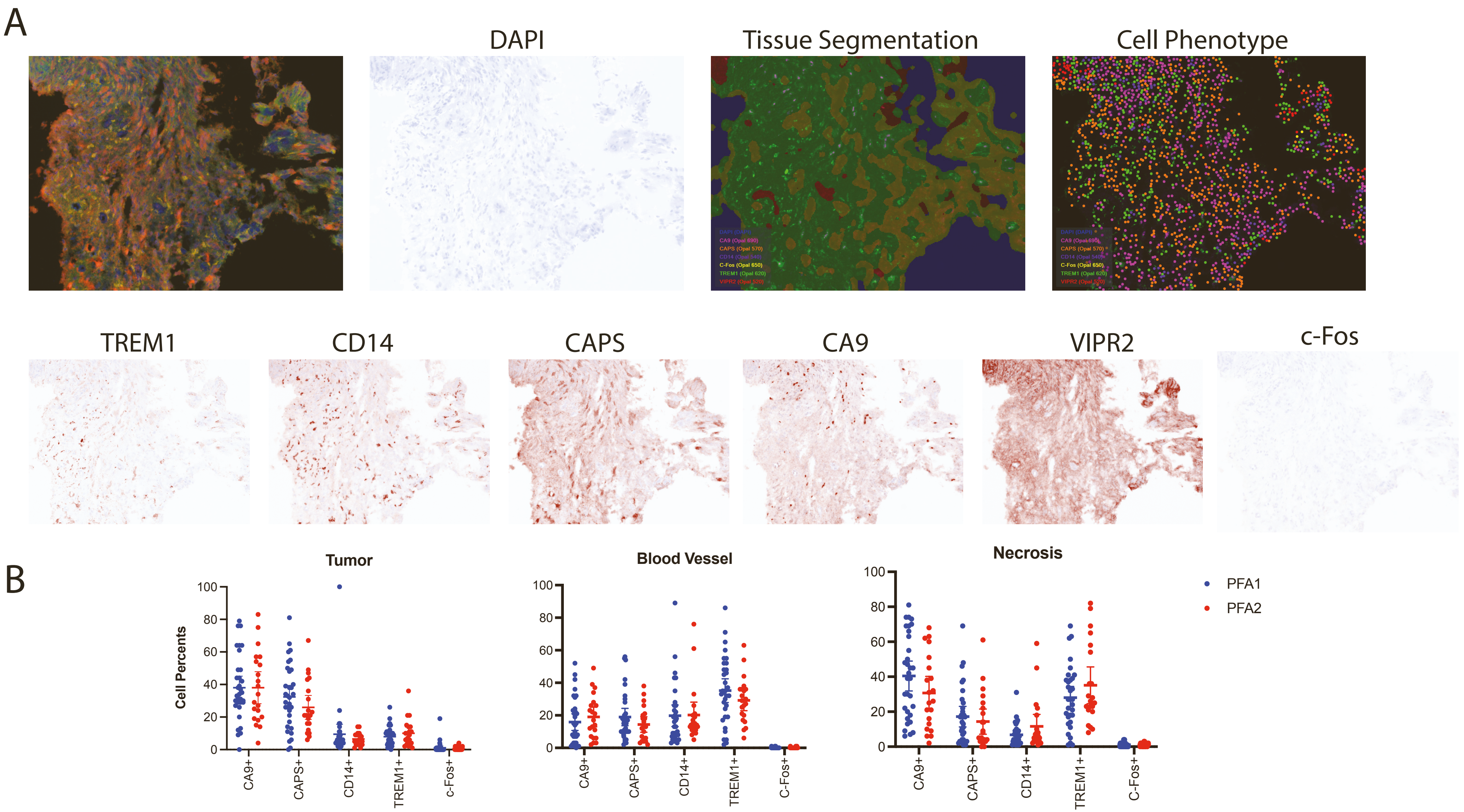

Figure S1. Immunofluorescent imaging panel vHu115 analysis strategy, related to *Quantification of multispectral immunofluorescent imaging* in Star Methods and Figure 4. A. Spectral unmixing of the EPN tissue scanned on Vectra 3.0 imager following staining with vHu115 IF panel. DAPI staining and matched H&E were used to identify tumor features (tumor, necrosis and blood vessels). Fifteen images were used to train the InForm software to segment tissue and 93% agreement was achieved. Cell phenotype were identified using single antibody patholo-gy views and approximately 600 cells were used to train the software algorithm. Cell phenotype was performed in batch analysis using InForm and the algorithm applied to the entire dataset. PhenoptR was used to consolidate the data. B. Consolidated data stratified by PFA subgroup reveals no differences in cell phenotypes.

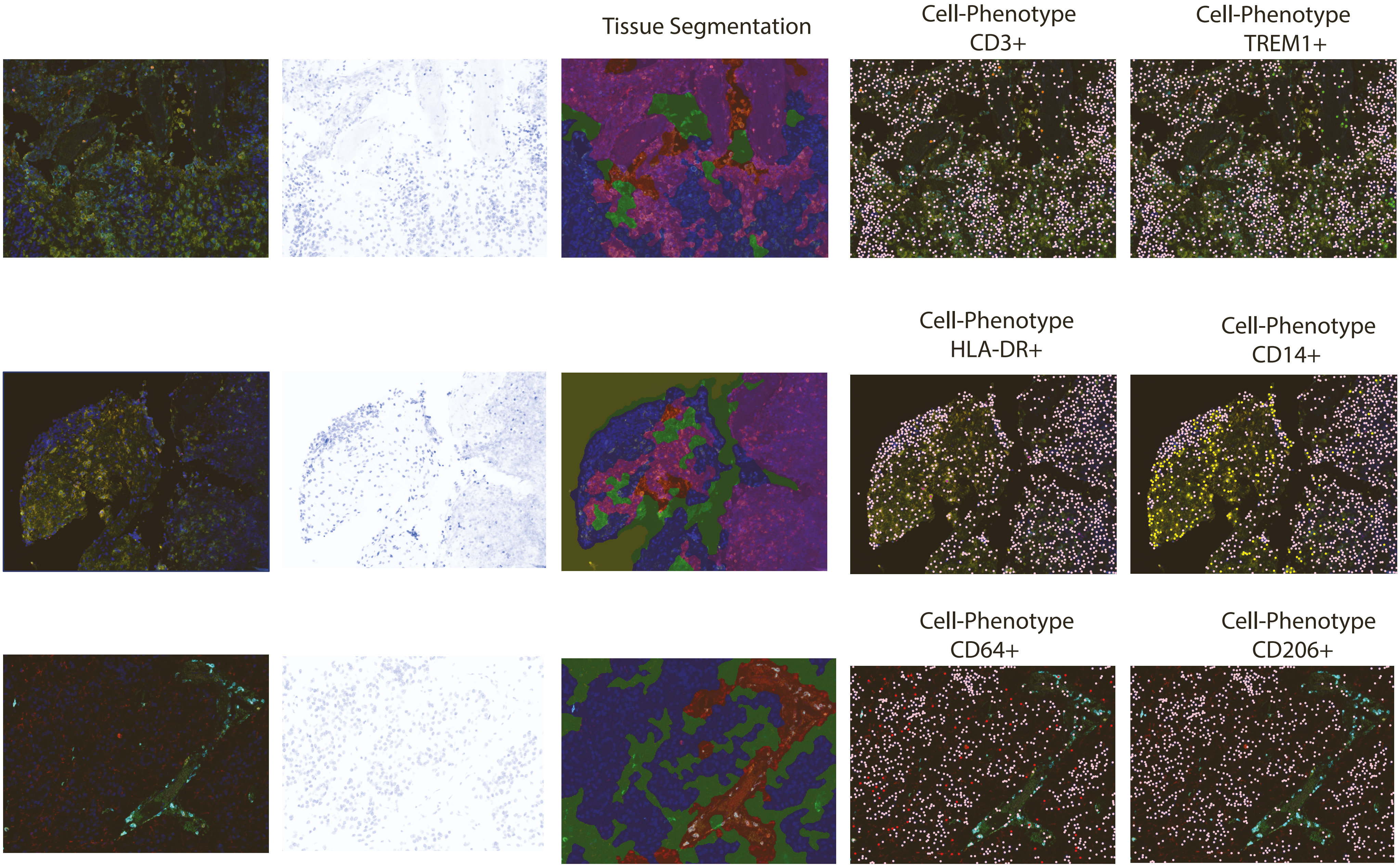

Figure S2. Immunofluorescent imaging panel vHu116 analysis strategy related to *Quantification of multispectral immunofluorescent imaging* in Star Methods and Figure 3. Spectral unmixing of the EPN tissue scanned on Vectra 3.0 image following staining with vHu116 IF panel. DAPI staining was used to identify tumor features. One image per MSI was used to train the InForm software to segment tissue and 91% agreement was achieved. Cell phenotypes were identified and exported as single antibody algorithm and applied as batch analyses across all MSIs for each project. The data was then merged and analysed using PhenoptR and PhenoptR reports. In the training images cells marked with a pink spot were considered negative. CD3+ cells are marked in orange, TREM1+ in green, HLA-DR+ in pink, CD14+ in yellow, CD64+ in red and CD206+ in blue.

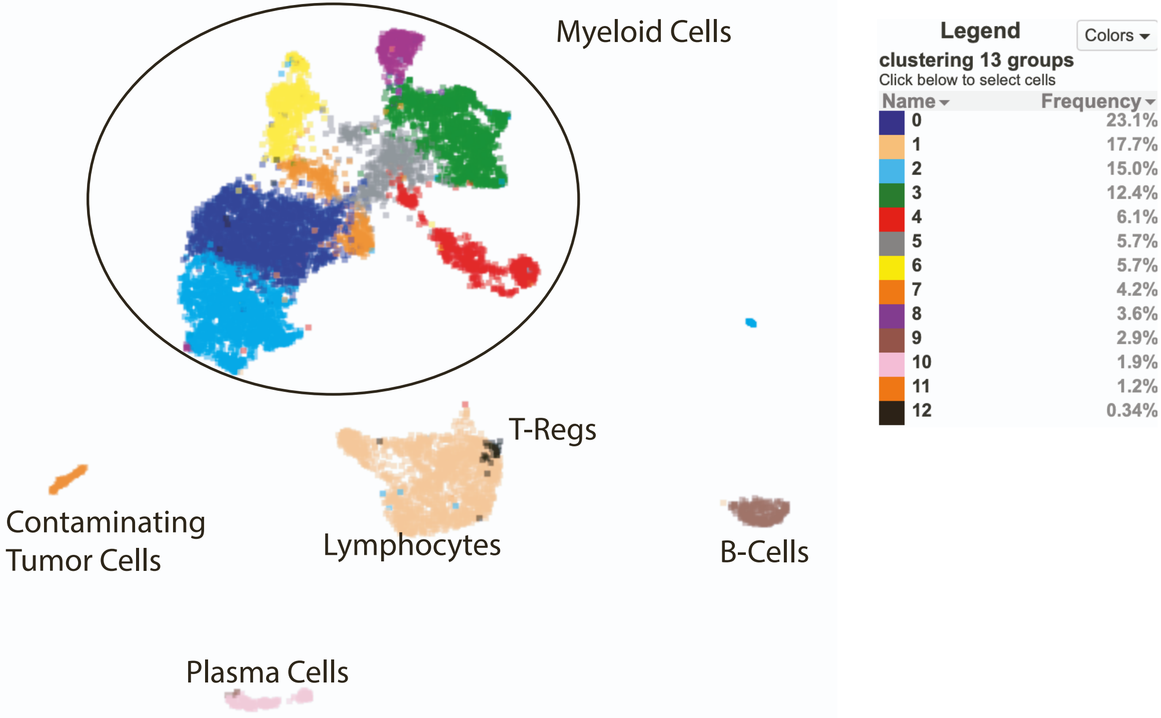

Figure S3. Single-cell RNA sequencing identified immune cell infiltration in pediatric ependymoma, related to Figure 1. UMAP visualization of the cellular subpopulations identified as immune cells using transcriptomic profiling. Thirteen unique populations were identified including; myeloid lineage, lymphocytes, B-cells, plasma cells and an unknown population.

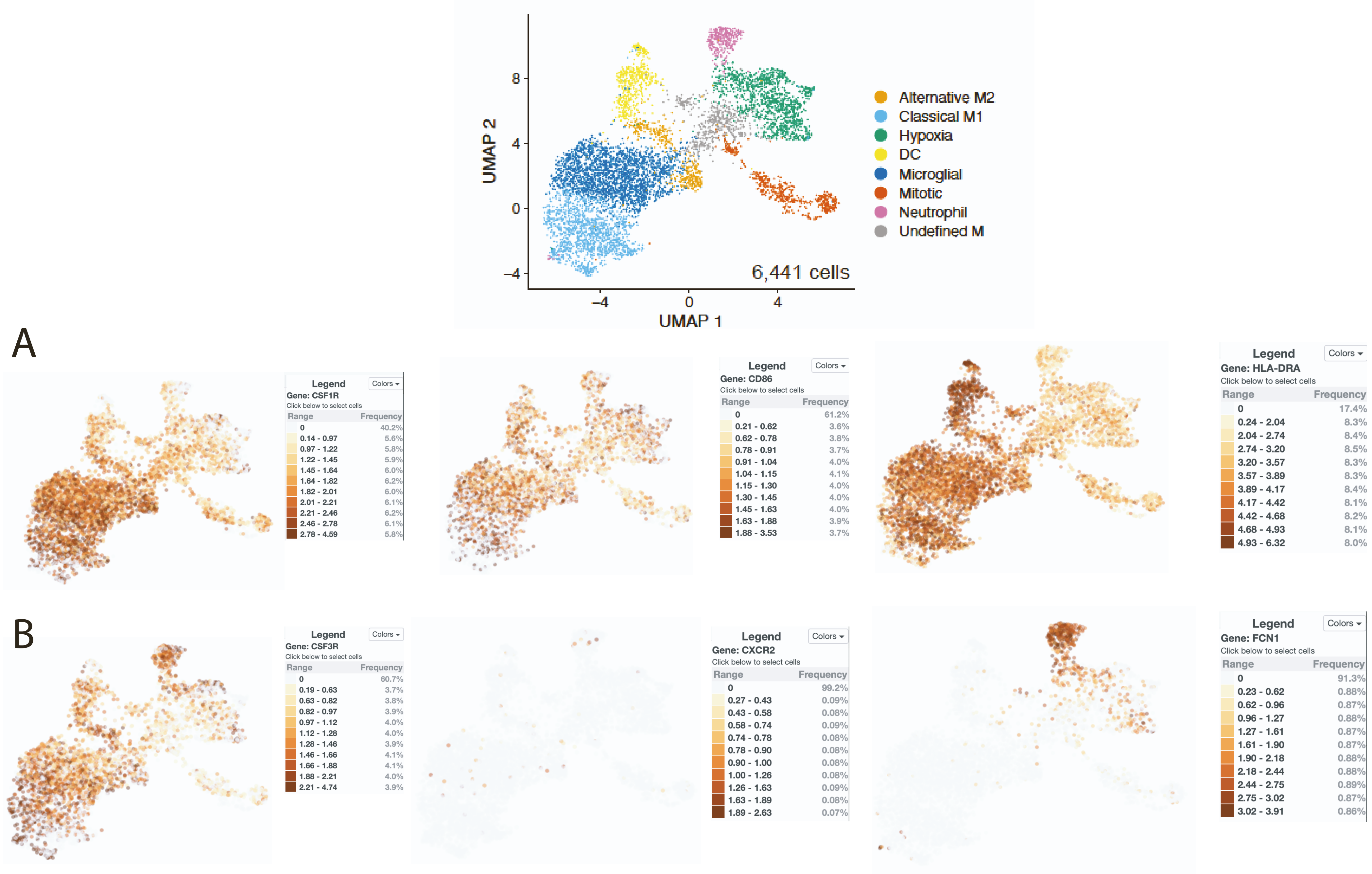

Figure S4: Common gene expression markers from perpherial blood, related to Figure 1 and Table S2. A. Common macrophage gene expression in ependymoma infiltrating myeloid cells. CSFR1, CD86 and HLA-DR, while higher expressed in microglial and classical myeloid cells, are also expressed by hypoxia myeloid cells, in particular CD86. B. Common neutrophil genes expression, CSF3R and FCN1, are highly enriched in the neutrophil subpopulation, with some expression in hypoxia myeloid, classical myeloid cells and microglial subpopulations. CXCR2 had dim expression in all subpopulations.

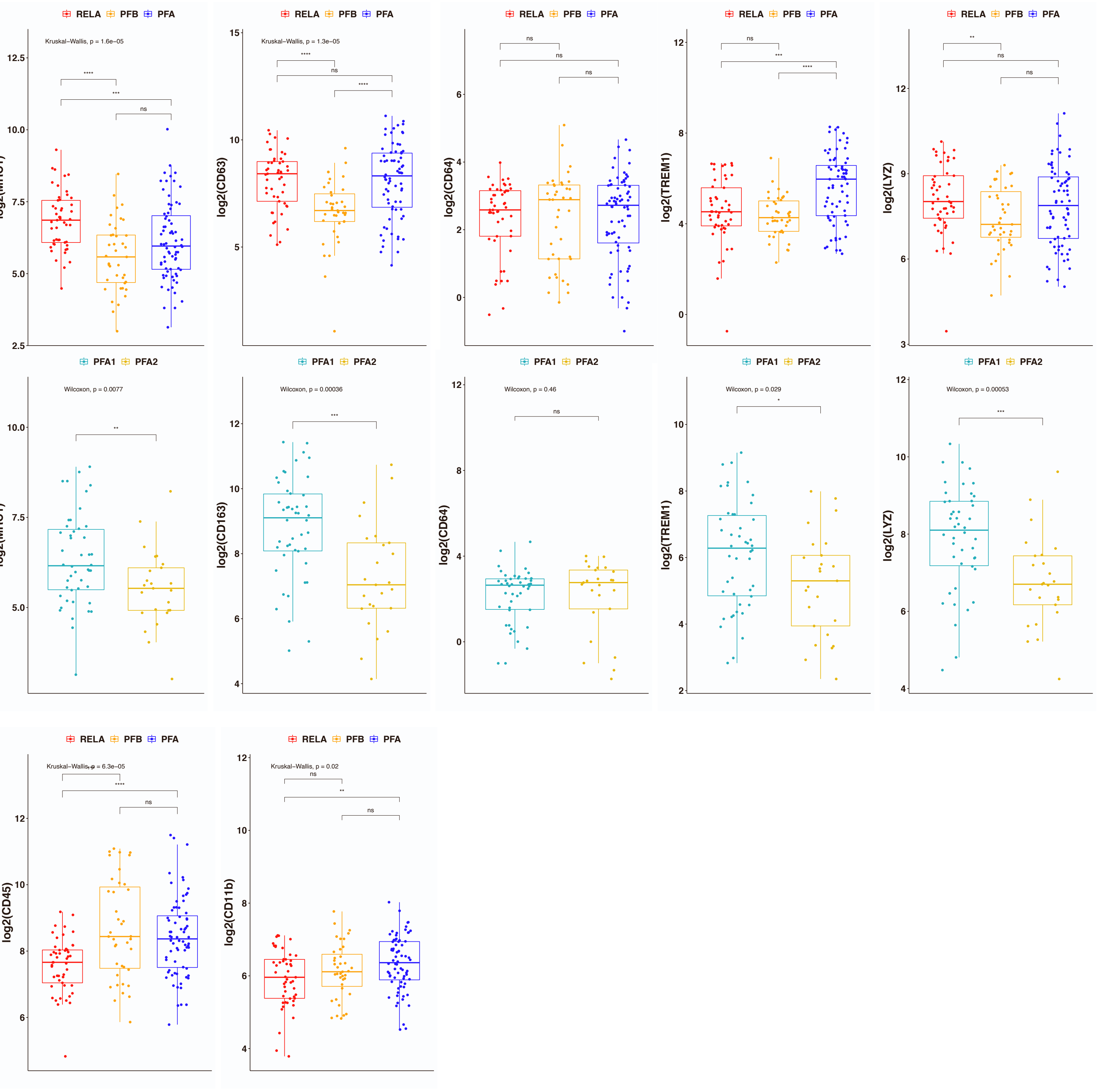

Figure S5. Gene expression levels of myeloid subpopulation genes using Cibersort analysis on bulk tumor microarray and RNAseq data, related to Figure 1, Figure 2 and Table S2. Summary of individual gene of interest data across the Heidelberg gene expression dataset (GSE64415) for molecular groups PFA, PFB and RELA and the Heidelberg, Denver and St. Jude's gene expression datasets for PFA1 and PFA2. Statistical analysis conducted via R2 (https://r2.amc.nl) and the R statistical environment. PFA samples from St. Jude, Heidelberg and Denver underwent clustering which confirmed that tumors did not show any center specific clustering indicating no evidence of batch effects across centers.

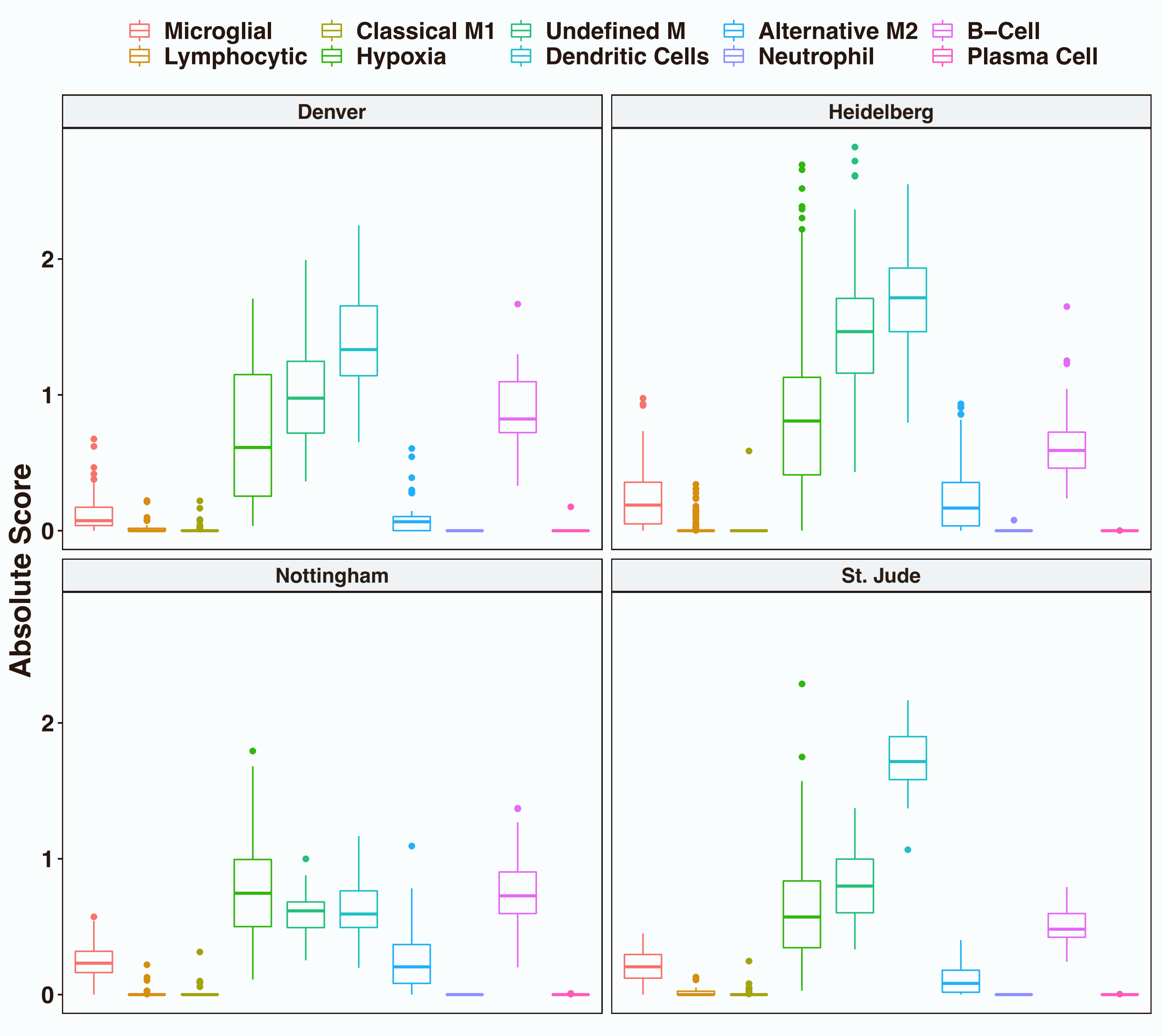

Figure S6. Summary of absolute Cibersort scores for each publically accessable bulk tumor micrarray and RNAseq dataset, related to Figure 1, Figure 2 and Table S2 Analysis illustrating the distribution of immune subpopulations in datasets originating from Denver (n=46), Heidelberg (n=160), Nottingham (n=54) and St. Jude (n=39). Absolute scores for the groups were then merged for downstream analysis given similarity of patterns of subpopulations.

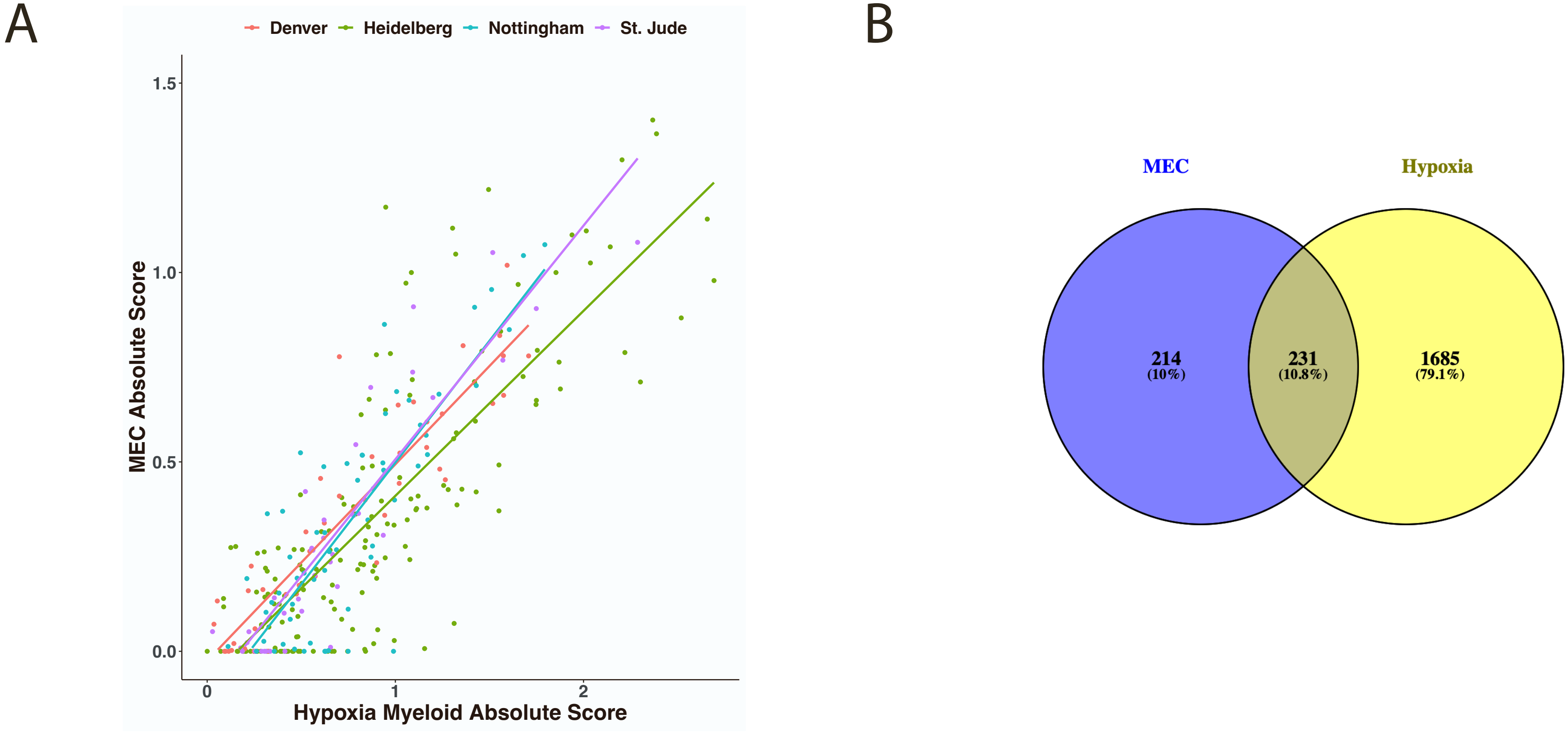

Figure S7. Association between MEC tumor cell gene expression profile and hypoxia myeloid cell gene expression profile using Cibersort analysis of bulk tumor microarray and RNAseq datasets, related to Figure 4 and Table S6. A. Correlation of Cibersort scores between the MEC tumor cell phenotype and the hypoxia myeloid phenotype. These two subpopulations were highly positively correlated in all four datasets with a combined Pearson's r value of 0.83 ($p<0.0001$, 95% CI 0.79-0.86). For each individual dataset (Denver, Nottingham, St. Jude, Heidelberg) the r values were 0.91, 0.83, 0.82 and 0.91 respectively, all with $p<0.0001$.Shared gene expression between mesenchymal ependymal cells (MEC) and Hypoxia myeloid cells. B. Venn diagram produced using Venny 2.1 online software tool. https://bioinfogp.cnb.csic.es/tools/venny/index.html

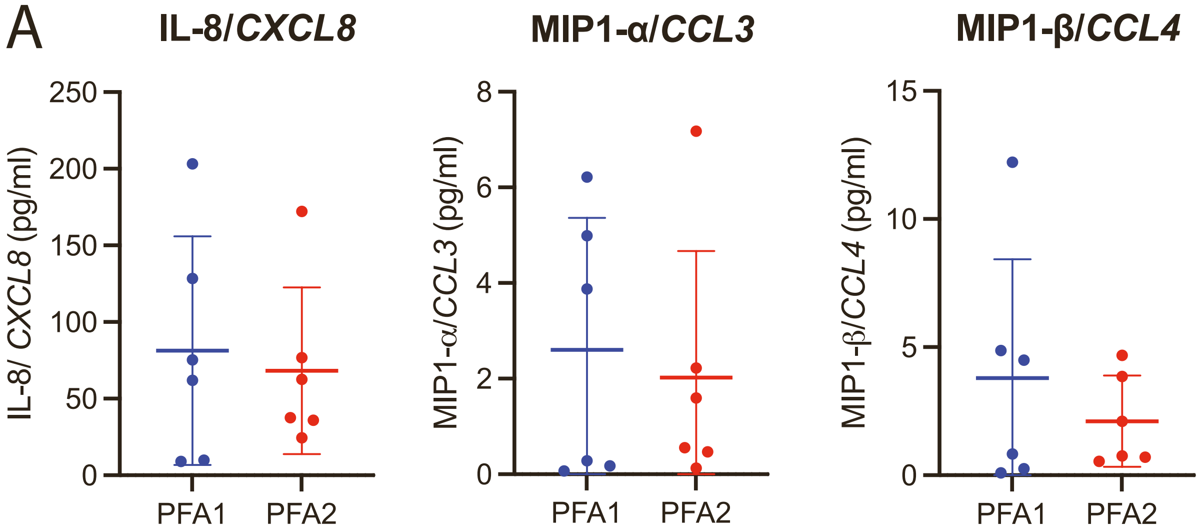

Figure S8. Secretion of cytokines from flow sorted EPN tumor inflitrating myeloid cells, related to Figure 5. A. Dissagregated tumor samples (n=6 PFA1 and n=6 PFA2 were thawed and stained with antibodies for human CD45 and CD11b. CD45+CD11b+ myeloid cells were isolated using flow cytometry and incubated in serum free media for 72 hours at 37 degC and 5% O2. Media was collected from cells and cytokines concentrations were measured using Milliplex Magnetic Bead 48-Plex kit.
